# Allowing for uncertainty due to missing and LOCF imputed outcomes in meta‐analysis

**DOI:** 10.1002/sim.8009

**Published:** 2018-10-22

**Authors:** Dimitris Mavridis, Georgia Salanti, Toshi A. Furukawa, Andrea Cipriani, Anna Chaimani, Ian R. White

**Affiliations:** ^1^ Department of Primary Education University of Ioannina Ioannina Greece; ^2^ Institute of Social and Preventive Medicine University of Bern Bern Switzerland; ^3^ Department of Health Promotion and Human Behavior Kyoto University Graduate School of Medicine/School of Public Health Kyoto Japan; ^4^ Department of Clinical Epidemiology Kyoto University Graduate School of Medicine/School of Public Health Kyoto Japan; ^5^ Department of Psychiatry University of Oxford Oxford UK; ^6^ Oxford Health NHS Foundation Trust Oxford UK; ^7^ Centre de Recherche Épidémiologie et Statistique Sorbonne Paris Cité (CRESS‐UMR1153) Inserm/Université Paris Descartes Paris France; ^8^ MRC Clinical Trials Unit University College London London UK

**Keywords:** expert opinion, informatively missing, last observation carried forward, pattern mixture model, sensitivity analysis

## Abstract

The use of the last observation carried forward (LOCF) method for imputing missing outcome data in randomized clinical trials has been much criticized and its shortcomings are well understood. However, only recently have published studies widely started using more appropriate imputation methods. Consequently, meta‐analyses often include several studies reporting their results according to LOCF. The results from such meta‐analyses are potentially biased and overprecise. We develop methods for estimating summary treatment effects for continuous outcomes in the presence of both missing and LOCF‐imputed outcome data. Our target is the treatment effect if complete follow‐up was obtained even if some participants drop out from the protocol treatment. We extend a previously developed meta‐analysis model, which accounts for the uncertainty due to missing outcome data via an informative missingness parameter. The extended model includes an extra parameter that reflects the level of prior confidence in the appropriateness of the LOCF imputation scheme. Neither parameter can be informed by the data and we resort to expert opinion and sensitivity analysis. We illustrate the methodology using two meta‐analyses of pharmacological interventions for depression.

## 1 INTRODUCTION

Missing data in clinical trials pervade all fields of medicine and may compromise the validity of inferences even from well‐designed randomized controlled trials.^1^ Trials usually follow patients over time and take measurements at several time points. Many participants drop out from follow‐up before the end of the study but have their outcomes reported at intermediate time points. Our target is the treatment effect if complete follow‐up was obtained, even if some participants discontinue the protocol treatment. Of course, discontinuing treatment is indicative of how effective and acceptable a treatment is, but ideally, the target in a randomized controlled trial is to take measurements and calculate an effect size at the end of the trial in order to abide by the intention‐to‐treat principle.

To achieve this, some imputation method is needed. A standard methodology in many clinical fields for imputing incomplete longitudinal data sets is the last observation carried forward (LOCF) method: The missing outcome is replaced by the last observed value. Missing data are particularly evident in mental health trials where dropout rates may exceed 50%^2^ and the LOCF method is commonly applied.^3^

An LOCF analysis is valid for estimating the treatment effect under very restrictive and usually unrealistic assumptions. In medical fields, disease progression is a definite feature and patients are expected to deteriorate over time, eg, in dementia,^4^ assuming no progression after dropout is expected to give biased results. In such a case, a LOCF analysis would give overly optimistic results for both groups; if participants in the treatment group leave earlier (ie, due to adverse events) or more frequently, then results would favor the treatment group. However, in depression and psychosis trials, we expect participants to improve over time and an early stop may give conservative results if participants in the experimental treatment drop out earlier because of adverse events.

Establishing a treatment effect based on an analysis that is clearly conservative represents compelling evidence of efficacy from a regulatory perspective.^1^ However, LOCF may induce bias in unpredictable ways, so treatment effects estimated using these assumptions are not necessarily conservative. LOCF (and other single‐imputation methods) does not propagate imputation uncertainty and leads to an underestimation of standard errors, which, in turn, increases the likelihood of finding a false positive result.

Although more appropriate methods have been proposed and adopted in new trials, older trials included in systematic reviews and meta‐analyses often use LOCF.^5^ A recent study showed that more than 75% of meta‐analyses in mental health contained studies that had LOCF imputed outcomes.^3^ The availability of individual participant data is rare and, as a result, meta‐analyses are not able to use appropriate imputation methods (eg, multiple imputation, likelihood methods) within each study. In this paper we focus on meta‐analysis with aggregate data (AD) and provide methods to reanalyze any study in an AD meta‐analysis whose reporting used the most common single‐imputation methods. In these reanalyses, we make a range of assumptions about the missing data. We use the term ‘LOCF analysis’ to refer to a synthesis of the reported outcome data from completers with LOCF‐imputed outcome values.

If studies take one single measurement at the end of the trial, then the complete case analysis would be valid under the missing‐at‐random (MAR) assumption: Missingness is conditionally independent of the outcome given any predictor. In either case (multiple or one final measurement), the probability of missingness may depend on unobserved characteristics such as the value of the missing outcome. In this case, data are missing not at random (MNAR). Patients in the treatment group may leave earlier because of adverse events, or patients randomized to a placebo group or a suboptimal treatment may leave earlier because of improvement and an LOCF analysis would give a biased treatment effect.

Methods to account for missing outcome data in AD meta‐analysis have been previously developed.^6^ They are primarily based on informative missingness parameters; parameters that relate the observed outcomes in completers to the assumed missing outcomes. White et al presented a pattern mixture model for handling dichotomous missing outcomes in which the degree of departure from the MAR assumption is quantified by the informative missingness odds ratio; this is defined as the ratio of the odds of the outcome in the missing participants to the odds of the outcome in the completers.^7^, ^8^ Mavridis et al extended the approach to missing continuous outcomes and to network meta‐analysis by quantifying the degree of departure from a MAR assumption using various informative missingness parameters such as an informative missingness difference of means (IMDoM, the difference in mean value of outcome in the missing participants and completers).^9^

Little work has been done, however, to account for uncertainty in data that have been imputed using LOCF. Dimitrakopoulou et al considered a sensitivity analysis by decomposing the probability of an unobserved successful outcome assuming various prior distributions for the sensitivity and specificity of the LOCF imputation.^10^ Here, we extend our previous work on AD meta‐analysis with continuous outcomes to account not only for missing outcome data but also for outcomes that have been imputed using LOCF.

We propose a pattern‐mixture model that allows us not only to consider LOCF as a special case but also to assume LOCF with some uncertainty introduced for the imputed values. Hence, we may get LOCF estimates with increased uncertainty reflecting the facts that we made an assumption that may not be true and that imputed data should not be treated as if they had been actually observed. The suggested model uses expert opinion to correct for bias. If expert opinion is not available, we can employ a sensitivity analysis to explore how robust results are to departures from the LOCF assumptions. The methods potentially work for the most common single‐imputation method, but we describe them for LOCF as this is the commonest and we describe other single‐imputation methods in the discussion.

This paper is organized as follows. In Section 2, we present two data sets from a large network of depression trials.^11^ In Section 3, we define the model. In Section 4, we discuss how we can inform the informative missingness parameters of the model, and in Section 5, we illustrate the methodology using the data sets presented in Section 2. We conclude with a discussion in Section 6.

## 2 MOTIVATING EXAMPLES

**Figure 1:**
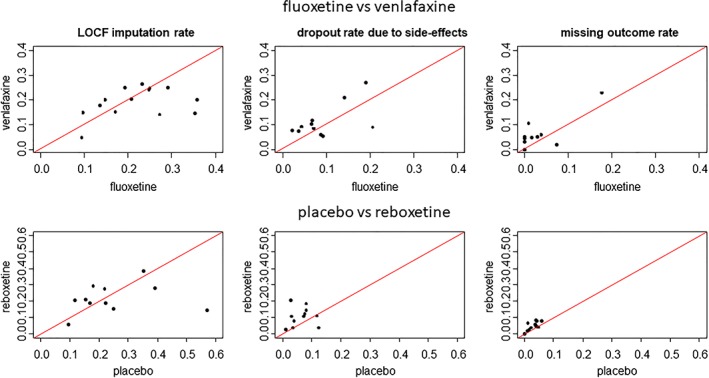
Proportion of participants (left side) who are imputed using the last observation carried forward (LOCF), (center) who drop out because of side effects, and (right side) who have missing outcomes (top row) for fluoxetine vs venlafaxine and (bottom row) for placebo vs reboxetine [Colour figure can be viewed at wileyonlinelibrary.com]

We use two data sets to illustrate the suggested methodology. The first data set (Table 1) consists of 14 studies comparing fluoxetine and venlafaxine, whereas the second one (Table 2) consists of 11 studies comparing reboxetine with placebo. Both comparisons are taken from a large network of depression trials.^11^ In both data sets, the outcome is the reduction in symptoms of depression in the Hamilton depression scale. Figure 1 shows the proportions of participants who are imputed using LOCF, drop out from follow‐up because of side effects, and have missing outcomes, for fluoxetine and venlafaxine (graphs on top row) and for reboxetine and placebo (graphs on bottom row).

Dropout for side effects and dropout before providing any measurement (missing outcomes) are more likely in the experimental groups (venlafaxine and reboxetine). The overall LOCF imputation rate is more balanced. We conjecture that participants randomized to the experimental groups tend to leave the studies early because of side effects, whereas those randomized to the control groups tend to leave somewhat later because of lack of efficacy. The inequalities between missing/imputation rates raise concerns that data are likely to be MNAR and study effects are potentially biased.

It is interesting that the three studies that provide an effect from both a complete case and an LOCF analysis (that includes both completers and imputed outcomes) show larger and less precise effect estimates for the former (Table 1). For example, the study of Sheehan et al shows a very large effect in the complete case analysis, ie, −0.61 (95% confidence interval, −0.95 to −0.28), and a much smaller effect in the LOCF analysis, ie, −0.27 (95% confidence interval, −0.55 to 0.02).

**Table 1:** Sample size, mean value, and standard deviation for completers plus imputed and completers for the comparison fluoxetine vs venlafaxine. The number of LOCF‐imputed and missing outcomes, and SMDs from the complete case analysis and the LOCF analysis are also given

id	Treatment	LOCF Analysis					Complete Case Analysis				
		SMD	Sample	Mean	SD	LOCF, %	SMD	Sample	Mean	SD	Missing, %
		(95% CI)	Size				(95% CI)	Size			
Clerc	Fluoxetine	−0.58	34	17,40	11,60	12(35%)	NA	22	NA	NA	0(0%)
1994	Venlafaxine	(−1.07,−0.09)	33	11,00	10,30	5(15%)	28	NA	NA	1(3%)
Dierick	Fluoxetine	−0.18	161	12,40	8,88	40(25%)	NA	121	NA	NA	0(0%)
1996	Venlafaxine	(−0.40,0.04)	153	10,70	9,90	38(25%)	115	NA	NA	0(0%)
Tylee	Fluoxetine	NA	NA	NA	NA	0(0%)	−0.08	140	11,24	13,34	30(18%)
1997	Venlafaxine	NA	NA	NA	0(0%)	(−0.32,0.16)	132	10,19	13,34	39(23%)
Costaesilva	Fluoxetine	−0.05	186	10,20	7,52	18(10%)	NA	168	NA	NA	0(0%)
1998	Venlafaxine	(−0.25,0.15)	196	9,80	7,52	29(15%)	167	NA	NA	0(0%)
Alves	Fluoxetine	−0.26	47	10,55	8,59	9(19%)	NA	38	NA	NA	0(0%)
1999	Venlafaxine	(−0.68,0.17)	40	8,31	8,59	10(25%)	30	NA	NA	0(0%)
Rudolph	Fluoxetine	−0.21	103	14,20	8,19	28(27%)	−0.27	75	12,80	9,00	0(0%)
1999	Venlafaxine	(−0.49,0.07)	95	12,50	8,10	14(15%)	(−0.58,0.05)	81	10,40	9,00	5(5%)
Silverstone	Fluoxetine	−0.05	119	13,40	7,94	30(25%)	NA	89	NA	NA	2(2%)
1999	Venlafaxine	(−0.30,0.20)	122	13,00	7,90	31(25%)	91	NA	NA	6(5%)
Tzanakaki	Fluoxetine	−0.09	50	12,50	8,59	8(16%)	−0.18	42	11,10	9,00	4(7%)
2000	Venlafaxine	(−0.48,0.29))	54	11,70	8,59	11(20%)	(−0.60,0.25)	43	9,50	9,00	1(2%)
Schatzberg	Fluoxetine	−0.17	99	16,30	8,59	29(29%)	NA	70	NA	NA	1(1%)
2006	Venlafaxine	(−0.46,0.11)	93	14,80	8,59	26(28%)	67	NA	NA	11(11%)
Nemeroff	Fluoxetine	−0.20	100	13,90	8,59	14(14%)	NA	86	NA	NA	4(4%)
2007	Venlafaxine	(−0.48,0.08)	96	12,20	8,59	18(19%)	78	NA	NA	6(6%)
Keller	Fluoxetine	0.04	266	8,90	6,52	47(18%)	NA	220	NA	NA	8(3%)
2007	Venlafaxine	(−0.10,0.18)	781	9,20	8,38	124(16%)	656	NA	NA	41(5%)
Sheehan	Fluoxetine	−0.27	99	18,09	8,89	23(23%)	−0.61	76	17,03	8,81	0(0%)
2009	Venlafaxine	(−0.55,0.02)	91	15,59	9,81	25(28%)	(−0.95,−0.28)	66	11,85	7,92	4(4%)
Heller	Venlafaxine	−0.28	15	8,86	4,50	3(20%)	NA	12	NA	NA	0(0%)
2009	Fluoxetine	(−1.01,0.45)	14	10,15	4,52	5(36%)		9	NA	NA	0(0%)
Chang	Fluoxetine	0.09	58	8,00	7,70	12(21%)	NA	46	NA	NA	0(0%)
2015	Venlafaxine	(−0.28,0.46)	54	8,70	8,30	11(20%)		43	NA	NA	0(0%)
POOLED RESULTS		−0.13	Heterogeneity SD = 0.09				−0.28	Heterogeneity SD = 0.18			
RANDOM EFFECTS		(−0.20,−0.05)					(−0.51,−0.04)				

Abbreviations: CI, confidence interval; LOCF, last observation carried forward; SD, standard deviation; SMD, standardized mean difference.

**Table 2:** Sample size, mean value, and standard deviation for completers plus imputed and completers for the comparison placebo vs reboxetine. The number of LOCF‐imputed and missing outcomes is also given

id	Treatment	LOCF Analysis					Completers				
		SMD	Sample	Mean	SD	LOCF, %	SMD	Sample	Mean	SD	Missing, %
		(95% CI)	Size				(95% CI)	Size			
Versiani2000	Reboxetine	−1.42	28	12,60	10,30	4(14%)	−0.70	22	10,10	8,20	0(0%)
(Study 091)	Placebo	(−2.01,−0.84)	28	29,50	13,30	16(57%)	(−1.47,0.07)	10	16,30	10,20	0(0%)
Study 032a	Reboxetine	0.12	22	17,18	4,75	7(32%)	0.22	17	16,59	4,73	2(12%)
(CTN032‐	Placebo	(−0.46,0.69)	25	16,6	5,14	5(20%)	(−0.42,0.87)	21	15,52	4,78	1(5%)
FCE20124)											
Study 015	Reboxetine	−0.19	110	14,04	9,22	23(21%)	−0.24	89	11,26	7,17	2(2%)
	Placebo	(−0.45,0.08)	111	15,8	9,58	26(23%)	(−0.54,0.06)	87	13,08	8,06	1(1%)
Bosc1997a	Reboxetine	−0.57	126	−13,45	8,45	38(30%)	NA	88	NA	NA	0(0%)
(Study 014 ‐	Placebo	(−0.82,−0.32)	128	−8,64	8,45	52(41%)		76	NA	NA	0(0%)
Andreoli2002)											
Ban1998	Reboxetine	−0.61	81	11,60	7,64	8(10%)	−0.69	73	10,40	6,32	3 (4%)
(Study 008)	Placebo	(−0.93,−0.30)	83	16,68	8,87	10 (12%)	(−1.02,−0.36)	73	15,52	8,37	2(3%)
Study 049	Reboxetine	−0.18	101	−9,30	5,44	37(37%)	−0.19	71	−11,40	10,00	6(8%)
	Placebo	(−0.46,0.09)	101	−8,30	5,44	23(23%)	(−0.51,0.13)	81	−9,50	10,00	4(5%)
Study 045	Reboxetine	0.21	174	−9,56	8,48	63(36%)	0.06	119	−13,32	9,96	12(10%)
	Placebo	(−0.05,0.46)	86	−11,30	8,45	20(23%)	(−0.24,0.36)	68	−13,90	10,00	1(1%)
Clayton2003	Reboxetine	0.04	144	−10,80	8,45	63(44%)	0.08	90	−13,30	10,00	6(7%)
(Study 050)	Placebo	(−0.20,0.27)	143	−11,10	8,45	60(42%)	(−0.21,0.37)	89	−14,10	10,00	7(8%)
M/2020/0046	Reboxetine	0	252	−11,50	8,45	67(27%)	−0.03	205	−12,70	10,00	13(6%)
(Study 046)	Placebo	(−0.18,0.18)	247	−11,50	8,45	40(16%)	(−0.22,0.16)	221	−12,40	10,00	10(5%)
M/2020/0047	Reboxetine	−0.13	238	−11,00	6,91	69(29%)	−0.10	189	−12,30	10,00	20(11%)
(Study 047)	Placebo	(−0.31,0.05)	239	−10,10	7,27	58(24%)	(−0.30,0.10)	200	−11,30	10,00	15(8%)
Studie009	Reboxetine	−0.08	24	14.38	8,94	6(25%)	−0.31	18	12.56	8,30	2(11%)
(CTN009‐	Placebo	(−0.65,0.49)	23	15.09	8,52	7(30%)	(−0.98,0.36)	17	15.12	8,28	1(6%)
FCE20124)											
POOLED RESULTS		−0.24	Heterogeneity SD = 0.29				−0.15	Heterogeneity SD = 0.17			
RANDOM EFFECTS		(−0.43,−0.05)					(−0.30,0.00)				

Abbreviations: CI, confidence interval; LOCF, last observation carried forward; SD, standard deviation; SMD, standardized mean difference.

The analysis of completers (using only four studies) gave a summary standardized mean difference (SMD) of −0.28 (95% confidence interval, −0.51 to −0.04) and a heterogeneity standard deviation (*τ* = 0.18), suggesting that there is a small difference between the two antidepressants. An analysis of the LOCF data gave a summary SMD of −0.13 (95% confidence interval, − 0.20 to −0.05) with *τ* = 0.09, drawing the same conclusions but with a more precise and less heterogeneous effect size. Both sources of data are likely to be biased, because the latter has used a single‐imputation method and because the former does not include more than two thirds of the studies and all the participants who dropped out.

In Table 2, all study‐specific SMDs are more precise in the LOCF analysis than in completers, although within‐study standard deviations are smaller in completers. This happens because sample size in the LOCF analysis is much bigger. The Versiani 2000 study had an imputation rate of 57% in the placebo group compared with a 14% rate in the experimental group. As a result, the LOCF analysis hardly showed a benefit in the placebo group and an SMD of −1.42 (95% confidence interval, −2.01 to −0.84) was computed. The corresponding SMD for the completers is −0.70 (95% confidence interval, −1.47 to 0.07). The analysis of completers gave a summary SMD of −0.15 (95% confidence interval, −0.30 to 0.00) and a heterogeneity standard deviation (*τ* = 0.17), suggesting that there is marginally not a statistically significant difference between the two antidepressants. An analysis of the LOCF data gave a summary SMD of −0.24 (95% confidence interval, − 0.43 to − 0.05) with heterogeneity *τ* = 0.29.

We see from these two examples that LOCF does not always give more conservative meta‐analytic results than completers analysis. Although LOCF is typically suggested as a conservative method, in the second example, all studies are more precise in the LOCF analysis compared with those in the complete‐case analysis. The LOCF pooled estimate, however, is less precise because heterogeneity is much larger in the LOCF analysis. Hence, the decrease in within‐study variations in the LOCF analysis brought an increase in between‐study variation.

## 3 METHODS

### 3.1 Notation and model definition

We divide all randomized individuals into three groups. *Completers* are those who completed the study providing outcome data at the end of the study. *Imputed* are those who did not complete the study but provided an outcome at an intermediate step and whose missing values at the end of the trial were imputed using LOCF (or another single‐imputation method). *Missing* are those who left the study without providing any outcome data. An analysis of the completers only is a complete case analysis. The *completers* and *imputed* together form the *reported* outcomes, and we refer to an analysis of these outcomes as an LOCF analysis.

In the notation, index *i* refers to study, *j* refers to study arm, and *k* refers to individuals. The notation involving *π* denotes population probabilities that a participant is of a particular type (completer/imputed/missing); *χ* and *σ* denote population outcome means and standard deviations, respectively; and *p*, *x*, *s* denote sample counterparts of these quantities.

Among participants randomized to arm *j* of study *i*, we count $n_{\mathrm{ij}}^{\mathrm{com}}$ completers, $n_{\mathrm{ij}}^{\mathrm{imp}}$ imputed, and $n_{\mathrm{ij}}^{\text{miss}}$ missing. Therefore, the fraction who reported at least one post‐baseline measurement during the study is $p_{\mathrm{ij}}^{\mathrm{rep}}=\frac{n_{\mathrm{ij}}^{\mathrm{com}}+n_{\mathrm{ij}}^{\mathrm{imp}}}{n_{\mathrm{ij}}^{\mathrm{com}}+n_{\mathrm{ij}}^{\mathrm{imp}}+n_{\mathrm{ij}}^{\text{miss}}}$ with complement $p_{\mathrm{ij}}^{\text{miss}}=1-p_{\mathrm{ij}}^{\mathrm{rep}}$. We use tilde throughout the manuscript to refer to quantities and estimates that have been potentially *contaminated* by the LOCF imputation. What we observe is ${\tilde{x}}_{\mathrm{ij}}^{\mathrm{rep}}$, the mean outcome for the completers and imputed participants. A thorough description of the model parameters is shown in Table 3.

**Table 3:** True parameters for study i and study group j

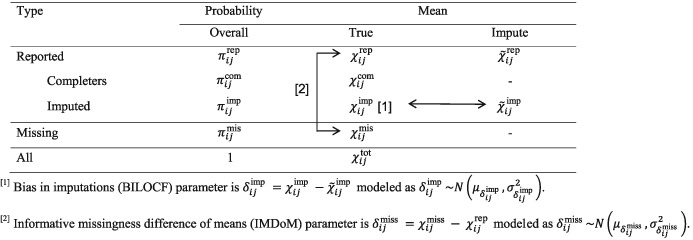

We define *Y*_*ijk*_ to be the true outcome of the *k*th individual at the end of the trial and and we define indicator variable *R*_*ijk*_ to be 1 in reported outcomes and 0 in missing outcomes, where

$$\begin{array}{c} P\left(R_{ijk}=1\right)=\pi_{ij}^{\text{rep}}\\ \begin{array}{c} E\left(Y_{ijk}|R_{ijk}=1\right)=\chi_{ij}^{\text{rep}}\\ E\left(Y_{ijk}|R_{ijk}=0\right)=\chi_{ij}^{\text{miss}}. \end{array} \end{array}$$

We then define $\chi_{\mathrm{ij}}^{\mathrm{com}}$ and $\;\chi_{\mathrm{ij}}^{\mathrm{imp}}$ as the true mean outcomes in completers and imputed participants, respectively. We also denote by $\pi_{\mathrm{ij}}^{*\mathrm{com}}$ and $\pi_{\mathrm{ij}}^{*\mathrm{imp}}$, with sample counterparts $p_{\mathrm{ij}}^{*\mathrm{com}}=\frac{n_{\mathrm{ij}}^{\mathrm{com}}}{n_{\mathrm{ij}}^{\mathrm{com}}+n_{\mathrm{ij}}^{\mathrm{imp}}}$ and $p_{\mathrm{ij}}^{*\mathrm{imp}}=\frac{n_{\mathrm{ij}}^{\mathrm{imp}}}{n_{\mathrm{ij}}^{\mathrm{com}}+n_{\mathrm{ij}}^{\mathrm{imp}}}$, the probabilities of an individual being a completer and imputed, respectively, conditional on having at least one outcome reported.

Thus, in those who had their outcome imputed, we distinguish the imputed outcome ${\tilde{Y}}_{\mathrm{ijk}}$ with expectation ${\tilde{\chi }}_{\mathrm{ij}}^{\mathrm{imp}}$ from the true unobserved outcome *Y*_*ijk*_ with expectation $\chi_{\mathrm{ij}}^{\mathrm{imp}}$. More details are given in Appendix A.

We aim to estimate the mean outcome *E*(*Y*_*ijk*_) and its variance var(*Y*_*ijk*_) for all individuals that were initially randomized to group *j* in study *i*. The former is expressed in the following equation:

(1) $$E\left(Y_{\mathrm{ijk}}\right)=\chi_{\mathrm{ij}}^{\mathrm{tot}}=\pi_{\mathrm{ij}}^{\mathrm{rep}}\chi_{\mathrm{ij}}^{\mathrm{rep}}+\pi_{\mathrm{ij}}^{\text{miss}}\chi_{\mathrm{ij}}^{\text{miss}}=\pi_{\mathrm{ij}}^{\mathrm{com}}\chi_{\mathrm{ij}}^{\mathrm{com}}+\pi_{\mathrm{ij}}^{\mathrm{imp}}\chi_{\mathrm{ij}}^{\mathrm{imp}}+\pi_{\mathrm{ij}}^{\text{miss}}\chi_{\mathrm{ij}}^{\text{miss}}.$$

The true outcome in the reported data, $\chi_{\mathrm{ij}}^{\mathrm{rep}}$, is not known. We define the expected mean value of the reported data using LOCF imputation as

(2) $${\tilde{\chi }}_{\mathrm{ij}}^{\mathrm{rep}}=\pi_{\mathrm{ij}}^{*\mathrm{com}}\chi_{\mathrm{ij}}^{\mathrm{com}}+\pi_{\mathrm{ij}}^{*\mathrm{imp}}{\tilde{\chi }}_{\mathrm{ij}}^{\mathrm{imp}}.$$

We develop a pattern mixture model as follows.

1. We estimate $\chi_{\mathrm{ij}}^{\mathrm{rep}}$ by associating it with the estimable parameter ${\tilde{\chi }}_{\mathrm{ij}}^{\mathrm{rep}}$ via an unidentified parameter using the methodology presented in Section 3.2.
2. We estimate the outcome $\chi_{\mathrm{ij}}^{\mathrm{tot}}$ as a mixture of $\chi_{\mathrm{ij}}^{\mathrm{rep}}$ and $\chi_{\mathrm{ij}}^{\text{miss}}$; see Equation (1). We associate $\chi_{\mathrm{ij}}^{\mathrm{tot}}$ with $\chi_{\mathrm{ij}}^{\mathrm{rep}}$ via an unidentified parameter using methodology presented in the IMDoM paper.^9^
3. We contrast $\chi_{\mathrm{ij}}^{\mathrm{tot}}$ across study arms within the same study to obtain effect sizes and their standard errors.
4. We synthesize effect sizes via inverse variance random‐effects meta‐analysis.^12^

### 3.2 Accounting for uncertainty and bias due to LOCF and missing outcome data

The aim here is to estimate the true outcome mean in participants who provided at least one outcome value. This is

(3) $$\chi_{\mathrm{ij}}^{\mathrm{rep}}=\pi_{\mathrm{ij}}^{*\mathrm{com}}\chi_{\mathrm{ij}}^{\mathrm{com}}+\pi_{\mathrm{ij}}^{*\mathrm{imp}}\chi_{\mathrm{ij}}^{\mathrm{imp}}.$$

However, only a sample estimate for ${\tilde{\chi }}_{\mathrm{ij}}^{\mathrm{rep}}$ is reported; see Equation (2).

To link $\chi_{\mathrm{ij}}^{\mathrm{rep}}$ to ${\tilde{\chi }}_{\mathrm{ij}}^{\mathrm{rep}}$, we introduce a new parameter, the bias in LOCF (BILOCF) parameter $\delta_{\mathrm{ij}}^{\mathrm{imp}}$, that quantifies the bias in the imputed values as the difference between the true outcome $\chi_{\mathrm{ij}}^{\mathrm{imp}}$ and the imputed outcome ${\tilde{\chi }}_{\mathrm{ij}}^{\mathrm{imp}}$ in patients who left the study early:

(4) $$\delta_{\mathrm{ij}}^{\mathrm{imp}}=\chi_{\mathrm{ij}}^{\mathrm{imp}}-{\tilde{\chi }}_{\mathrm{ij}}^{\mathrm{imp}}.$$

The BILOCF parameter is not estimable and we need to make assumptions about its value. We may consider a fixed value or a plausible range of values by assigning a distribution, eg, $\delta_{\mathrm{ij}}^{\mathrm{imp}}∼N\left(\mu_{\delta_{\mathrm{ij}}^{\mathrm{imp}}},\sigma_{\delta_{\mathrm{ij}}^{\mathrm{imp}}}^{2}\right)$, that would reflect our uncertainty about its true value. Letting $\delta_{\mathrm{ij}}^{\mathrm{imp}}=0$ is equivalent to an analysis of reported outcomes. In the examples considered in this manuscript, letting $\delta_{\mathrm{ij}}^{\mathrm{imp}}=0$ is equivalent to the LOCF analysis. We can acknowledge uncertainty about the correct analysis by letting $\mu_{\delta_{\mathrm{ij}}^{\mathrm{imp}}}=0$, meaning that our best guess is that those who dropped out neither improved nor deteriorated, and $\sigma_{\delta_{\mathrm{ij}}^{\mathrm{imp}}}^{2}>0$, expressing uncertainty about this guess. Effect estimates will be similar to the LOCF analysis but less precise. The methodology can be applied for other imputation schemes (eg, mean imputation).

From Equations (2), (3), and (4), it follows that

(5) $$\chi_{\mathrm{ij}}^{\mathrm{rep}}=\pi_{\mathrm{ij}}^{*\mathrm{com}}\chi_{\mathrm{ij}}^{\mathrm{com}}+\pi_{\mathrm{ij}}^{*\mathrm{imp}}\left(\delta_{\mathrm{ij}}^{\mathrm{imp}}+{\tilde{\chi }}_{\mathrm{ij}}^{\mathrm{imp}}\right)={\tilde{\chi }}_{\mathrm{ij}}^{\mathrm{rep}}+\pi_{\mathrm{ij}}^{*\mathrm{imp}}\delta_{\mathrm{ij}}^{\mathrm{imp}}.\;$$

We previously developed a model for missing outcome data that uses $\delta_{\mathrm{ij}}^{\text{miss}}$, an IMDoM^9^ parameter, that quantifies the difference in mean outcome between observed and missing participants:

(6) $$\delta_{\mathrm{ij}}^{\text{miss}}=\chi_{\mathrm{ij}}^{\text{miss}}-\chi_{\mathrm{ij}}^{\mathrm{rep}},$$

with $\delta_{\mathrm{ij}}^{\text{miss}}∼N\left(\mu_{\delta_{\mathrm{ij}}^{\text{miss}}},\sigma_{\delta_{\mathrm{ij}}^{\text{miss}}}^{2}\right)$. Again, we need to resort to assumptions to define this distribution.

The total outcome is

(7) $$\chi_{\mathrm{ij}}^{\mathrm{tot}}=\pi_{\mathrm{ij}}^{\mathrm{rep}}\chi_{\mathrm{ij}}^{\mathrm{rep}}+\pi_{\mathrm{ij}}^{\text{miss}}\chi_{\mathrm{ij}}^{\text{miss}}.$$

From Equations (5), (6), and (7), and assuming that $\delta_{\mathrm{ij}}^{\mathrm{imp}}$ and $\delta_{\mathrm{ij}}^{\text{miss}}$ are independent, we obtain

(8) $$\chi_{\mathrm{ij}}^{\mathrm{tot}}={\tilde{\chi }}_{\mathrm{ij}}^{\mathrm{rep}}+\pi_{\mathrm{ij}}^{*\mathrm{imp}}\delta_{\mathrm{ij}}^{\mathrm{imp}}+\pi_{\mathrm{ij}}^{\text{miss}}\delta_{\mathrm{ij}}^{\text{miss}}.\;$$

We now estimate these quantities from the data, which we write as expectations given the data. Using Equations (5) and (2), we obtain the *imputation‐adjusted outcome*

(9) $$E\left(\chi_{\mathrm{ij}}^{\mathrm{rep}}\right)=E\left(\pi_{\mathrm{ij}}^{*\mathrm{com}}\chi_{\mathrm{ij}}^{\mathrm{com}}+\pi_{\mathrm{ij}}^{*\mathrm{imp}}\left(\delta_{\mathrm{ij}}^{\mathrm{imp}}+{\tilde{\chi }}_{\mathrm{ij}}^{\mathrm{imp}}\right)|\text{data}\right)=\pi_{\mathrm{ij}}^{*\mathrm{com}}x_{\mathrm{ij}}^{\mathrm{com}}+\pi_{\mathrm{ij}}^{*\mathrm{imp}}{\tilde{\chi }}_{\mathrm{ij}}^{\mathrm{imp}}+\pi_{\mathrm{ij}}^{*\mathrm{imp}}\mu_{\delta_{\mathrm{ij}}^{\mathrm{imp}}}={\tilde{x}}_{\mathrm{ij}}^{\mathrm{rep}}+p_{\mathrm{ij}}^{*\mathrm{imp}}\mu_{\delta_{\mathrm{ij}}^{\mathrm{imp}}}.$$

We can also estimate an *imputation‐adjusted variance* for the mean outcome by using a Taylor‐series approximation and assuming that outcomes, probabilities of observing a pattern (completers, imputed, missing), and informative missingness parameters are uncorrelated as

(10) $$V\left(\chi_{\mathrm{ij}}^{\mathrm{rep}}\right)\approx V\left(\pi_{\mathrm{ij}}^{*\mathrm{com}}\chi_{\mathrm{ij}}^{\mathrm{com}}+\pi_{\mathrm{ij}}^{*\mathrm{imp}}\left(\delta_{\mathrm{ij}}^{\mathrm{imp}}+{\tilde{\chi }}_{\mathrm{ij}}^{\mathrm{imp}}\right)|\text{data}\right)=V\left({\tilde{x}}_{\mathrm{ij}}^{\mathrm{rep}}\right)+\frac{\left(\mu_{\delta_{\mathrm{ij}}^{\mathrm{imp}}}^{2}+\sigma_{\delta_{\mathrm{ij}}^{\mathrm{imp}}}^{2}\right)p_{\mathrm{ij}}^{*\mathrm{com}}p_{\mathrm{ij}}^{*\mathrm{imp}}}{n_{\mathrm{ij}}^{\mathrm{com}}+n_{\mathrm{ij}}^{\mathrm{imp}}}+p_{\mathrm{ij}}^{{*\mathrm{imp}}^{2}}\sigma_{\delta_{\mathrm{ij}}^{\mathrm{imp}}}^{2}.$$

Proofs are given in Appendix A.

If $p_{\mathrm{ij}}^{*\mathrm{com}}=1$ (all patients with intermediate measurements completed the study) or if $\delta_{\mathrm{ij}}^{\mathrm{imp}}=0\left(\mu_{\delta_{\mathrm{ij}}^{\mathrm{imp}}}=\sigma_{\delta_{\mathrm{ij}}^{\mathrm{imp}}}=0\right)$ (the imputation process is accurate without uncertainty), then $V\left(\chi_{\mathrm{ij}}^{\mathrm{rep}}\right)=V\left({\tilde{\chi }}_{\mathrm{ij}}^{\mathrm{rep}}\right)$. Otherwise, $V\left(\chi_{\mathrm{ij}}^{\mathrm{rep}}\right)>V\left({\tilde{\chi }}_{\mathrm{ij}}^{\mathrm{rep}}\right)$.

We can also let the BILOCF and IMDoM parameters be correlated. Mathematically, this is easily done (see Appendix B), but eliciting information about this correlation may be hard in practice.

Data inform directly ${\tilde{\chi }}_{\mathrm{ij}}^{\mathrm{rep}}$, $\pi_{\mathrm{ij}}^{*\mathrm{imp}}$, and $\pi_{\mathrm{ij}}^{\text{miss}}$, whereas the external assumptions inform the BILOCF $\left(\delta_{\mathrm{ij}}^{\mathrm{imp}}\right)\;$and the IMDoM $\left(\delta_{\mathrm{ij}}^{\text{miss}}\right)$. The expected value of the outcome conditional on the reported data is

(11) $$E\left(\chi_{\mathrm{ij}}^{\mathrm{tot}}|\text{data}\right)={\tilde{x}}_{\mathrm{ij}}^{\mathrm{rep}}+p_{\mathrm{ij}}^{*\mathrm{imp}}\mu_{\delta_{\mathrm{ij}}^{\mathrm{imp}}}+p_{\mathrm{ij}}^{\text{miss}}\mu_{\delta_{\mathrm{ij}}^{\text{miss}}}.$$

By taking the variance of Equation (8) conditional on the observed data and using Equation (10) to replace $V\left(\chi_{\mathrm{ij}}^{\mathrm{rep}}\right)$, we get

(12) $$V\left(\chi_{\mathrm{ij}}^{\mathrm{tot}}|\text{data}\right)\approx V\left({\tilde{x}}_{\mathrm{ij}}^{\mathrm{rep}}\right)+\frac{\left(\mu_{\delta_{\mathrm{ij}}^{\mathrm{imp}}}^{2}+\sigma_{\delta_{\mathrm{ij}}^{\mathrm{imp}}}^{2}\right)p_{\mathrm{ij}}^{*\mathrm{com}}p_{\mathrm{ij}}^{*\mathrm{imp}}}{n_{\mathrm{ij}}^{\mathrm{com}}+n_{\mathrm{ij}}^{\mathrm{imp}}}+{\left(p_{\mathrm{ij}}^{*\mathrm{imp}}\right)}^{2}\sigma_{\delta_{\mathrm{ij}}^{\mathrm{imp}}}^{2}+\frac{\left(\mu_{\delta_{\mathrm{ij}}^{\text{miss}}}^{2}+\sigma_{\delta_{\mathrm{ij}}^{\text{miss}}}^{2}\right)p_{\mathrm{ij}}^{\mathrm{rep}}p_{\mathrm{ij}}^{\text{miss}}}{n_{\mathrm{ij}}^{\mathrm{com}}+n_{\mathrm{ij}}^{\mathrm{imp}}+n_{\mathrm{ij}}^{\text{miss}}}+{\left(p_{\mathrm{ij}}^{\text{miss}}\right)}^{2}\sigma_{\delta_{\mathrm{ij}}^{\text{miss}}}^{2}.$$

It should be noted that participants drop out for various reasons. It may be unrealistic to assume the same BILOCF and IMDoM parameters ( $\delta_{\mathrm{ij}}^{\mathrm{imp}}$ and $\delta_{\mathrm{ij}}^{\text{miss}})$ across all imputed and missing participants, respectively (eg, for those who left because of lack of improvement and side effects). In Appendix D, we present how one can assume different scenarios according to the reasons for missingness and manipulate the aforementioned Equations accordingly by assuming different BILOCF and IMDoM parameters for the various types of missing participants. However, the numbers of participants left for any possible reason are rarely reported.

### 3.3 Estimating the effect size and its uncertainty for each trial

The unconditional means $\chi_{\mathrm{ij}}^{\mathrm{tot}}$ are contrasted to obtain the relative treatment effect in each study, which is defined as the difference

(13) $$\;\beta_{i}=f\left(\chi_{\mathrm{iT}}^{\mathrm{tot}}\right)-f\left(\chi_{\mathrm{iC}}^{\mathrm{tot}}\right),$$

where *j* = *C* and *j* = *T* refer to the control and treatment group and *f* is a link function that determines the effect measure. If *f* is the identity function, *f*(*u*) = *u*, then *β*_*i*_ is the mean difference (MD). If $f\left(u_{i}\right)=\frac{u_{i}}{S_{i}}$, where $S_{i}=\sqrt{\frac{\left(n_{\mathrm{iT}}-1\right)s_{\mathrm{iT}}^{2}+\left(n_{\mathrm{iC}}-1\right)s_{\mathrm{iC}}^{2}}{n_{\mathrm{iT}}+n_{\mathrm{iC}}-2}}$, we obtain the SMD. We show the working for the SMD in the Appendix. For MDs, it holds

(14) $$E\left(\beta_{i}|\text{data}\right)=E\left(\chi_{\mathrm{it}}^{\mathrm{tot}}|\text{data}\right)-E\left(\chi_{\mathrm{ic}}^{\mathrm{tot}}|\text{data}\right),$$

and applying Equation (11) in each arm of the right‐hand side of Equation (14), we obtain

(15) $$E\left[\;\beta_{i}|\text{data}\right]={\tilde{x}}_{\mathrm{iT}}^{\mathrm{rep}}+p_{\mathrm{iT}}^{*\mathrm{imp}}\mu_{\delta_{\mathrm{iT}}^{\mathrm{imp}}}+p_{\mathrm{iT}}^{\text{miss}}\mu_{\delta_{\mathrm{iT}}^{\text{miss}}}-{\tilde{x}}_{\mathrm{iC}}^{\mathrm{rep}}-p_{\mathrm{iC}}^{*\mathrm{imp}}\mu_{\delta_{\mathrm{iC}}^{\mathrm{imp}}}-p_{\mathrm{iC}}^{\text{miss}}\mu_{\delta_{\mathrm{iC}}^{\text{miss}}}.$$

We assume that the BILOCF and IMDoM parameters are correlated across arms with correlations $\rho_{\delta_{i}^{\mathrm{imp}}}$ and $\rho_{\delta_{i}^{\text{miss}}}$, respectively; that is, $\text{corr}\left(\delta_{\mathrm{iT}}^{\mathrm{imp}},\delta_{\mathrm{iC}}^{\mathrm{imp}}\right)=\rho_{\delta_{i}^{\mathrm{imp}}}$ and $\text{corr}\left(\delta_{\mathrm{iT}}^{\text{miss}},\delta_{\mathrm{iC}}^{\text{miss}}\right)=\rho_{\delta_{i}^{\text{miss}}}$. It follows that the variance of the MD is estimated as

(16) $$V\left(\;\beta_{i}|\text{data}\right)=V\left(\chi_{\mathrm{iT}}^{\mathrm{tot}}|\text{data}\right)+V\left(\chi_{\mathrm{iC}}^{\mathrm{tot}}|\text{data}\right)-2\rho_{\delta_{i}^{\mathrm{imp}}}\sigma_{\delta_{\mathrm{iT}}^{\mathrm{imp}}}\sigma_{\delta_{\mathrm{iC}}^{\mathrm{imp}}}p_{\mathrm{iT}}^{*\mathrm{imp}}p_{\mathrm{iC}}^{*\mathrm{imp}}-2\rho_{\delta_{i}^{\text{miss}}}\sigma_{\delta_{\mathrm{iT}}^{\text{miss}}}\sigma_{\delta_{\mathrm{iC}}^{\text{miss}}}p_{\mathrm{iT}}^{\text{miss}}p_{\mathrm{iC}}^{\text{miss}},$$

where $V\left(\chi_{\mathrm{iT}}^{\mathrm{tot}}|\text{data}\right)$ and $V\left(\chi_{\mathrm{iC}}^{\mathrm{tot}}|\text{data}\right)$ can be estimated from Equation (12). More information is given in Appendix B.

Then, we can conduct a meta‐analysis in two steps as follows.

- Compute study‐specific treatment effects and their variances from Equations (15) and (16).
- Conduct an inverse‐variance meta‐analysis.^12^

Alternatively, the model can be fit in a single one‐stage procedure,^8^ eg, in WinBUGS software.^13^

## 4 INFORMING THE MODEL PARAMETERS

The model presented in Section 3 is underidentified because the distributions of $\delta_{\mathrm{ij}}^{\mathrm{imp}}$ and $\delta_{\mathrm{ij}}^{\text{miss}}$ (BILOCF and IMDoM parameters) cannot be informed by the data. To inform these parameters, we can either use expert opinion, possibly informed by empirical data, eg, from studies with individual patient data to inform BILOCF, or conduct a sensitivity analysis assuming various distributions for $\delta_{\mathrm{ij}}^{\mathrm{imp}}$ and $\delta_{\mathrm{ij}}^{\text{miss}}$, to explore how robust results are to departures from the LOCF analysis.

Methods have been suggested in the literature^14^, ^15^ to elicit the distribution of $\delta_{\mathrm{ij}}^{\text{miss}}$. More details are given in Appendix C. We propose new methods to elicit the distribution of $\delta_{\mathrm{ij}}^{\mathrm{imp}}$. This involves experts' beliefs about those who dropped out of the study at an intermediate step and had their outcome imputed using LOCF. More specifically, we would like to know how different the imputed outcome is from that we would have observed had the individual stayed in the trial until its end.

We can use an expert opinion to inform the BILOCF parameter. Along with the number of imputed outcomes, we may inform the expert of the dropout times. Participants may have dropped out at different time points. Suppose that we measure reduction in symptoms in depression at 12 weeks using the Hamilton Rating Scale for Depression (HAMD) scale. Previous measurements exist for 4 and 8 weeks. We consulted two psychiatrists (AC and TF) with expertise in conducting depression trials with the aim to identify what information is important to deliver to the expert and to form appropriate questions for eliciting the parameters of interest ( $\delta_{\mathrm{ij}}^{\mathrm{imp}}$ and $\delta_{\mathrm{ij}}^{\text{miss}})$. We put forward the following question to the experts.

Participants randomized to fluoxetine who dropped out of follow‐up at 8 weeks after the onset of the treatment were observed at this point to have a mean score of 35 at the HAMD scale with 95% confidence interval [30‐40]. What is your prediction about their outcome at 12 weeks?

Then, we may repeat the question for measurements at a different time point (eg, we have measurements at 4 weeks) or for other antipsychotics (eg, venlafaxine) or placebo. Table 4 shows the responses of a hypothetical expert who believes that participants who left at 4 weeks would have improved considerably had they stayed in the study until its completion but participants who left at week 8 would not change at 12 weeks. Translating the answers from Table 4 into parameter values for the BILOCFs, we get approximately $\delta_{\mathrm{ij}}^{\mathrm{imp}}∼N\left(-6,3^{2}\right)$ for 4 weeks and $\delta_{\mathrm{ij}}^{\mathrm{imp}}∼N\left(0,6^{2}\right)$ for 8 weeks.

**Table 4:** Eliciting expert opinion to evaluate the differences in the outcomes between LOCF imputed participants and their true outcomes at the end of the trial. This table shows, for illustration purposes, a hypothetical example with the responses of an expert who believes that participants who left at 4 weeks would have reduced by many points in the Hamilton Rating Scale for Depression (HAMD) scale had they stayed in the trial, but those who left at 8 weeks would not change at the end of the trial

*Participants randomized to fluoxetine were observed to have a mean score of 35 at the HAMD scale with 95% confidence interval*									
*[30–40] at 4 and 8 weeks after onset of the treatment. What is your prediction about their outcome at 12 weeks?*									
**Left at 8 weeks**	If the patient stayed in the study, s/he would have been improved by								
	−12	−9	−6	−3	0	3	6	9	12
	around 23	around 26	around 29	around 32	around 35	around 38	around 41	around 44	around 47
**Your answers**	5	7	10	13	30	13	10	7	5
**Left at 4 weeks**	If the patient stayed in the study, s/he would have been improved by								
	−12	−9	−6	−3	0	3	6	9	12
	around 23	around 26	around 29	around 32	around 35	around 38	around 41	around 44	around 47
**Your answers**	10	20	40	20	10	0	0	0	0

We typically know neither the mean imputed outcome ( ${\tilde{\chi }}_{\mathrm{ij}}^{\mathrm{imp}})$ nor the time point participants dropped out from the study. The latter is very important. For two active antidepressants, the typical trajectory in the acute phase treatment of depression is that we have a large improvement in 2 to 4 weeks, a smaller one in 4 to 8 weeks, and then, the effect almost flattens out. For a comparison between an antidepressant and placebo, we would expect a small difference in the first 2 to 4 weeks and the largest difference would occur around 8 weeks, and then, the difference decreases. Hence, we may have different BILOCFs for different groups of participants (or even for different comparisons of interventions). We show how this can be implemented in Appendix D.

Ideally, we would like to provide the expert with the following information:

1. proportion of participants who were LOCF‐imputed;
2. mean outcome estimated from imputed participants $\left({\tilde{\chi }}_{\mathrm{ij}}^{\mathrm{rep}}\right)$ and its uncertainty; and
3. time of dropout (eg, 20% left before completion of eight weeks—usually not available in the absence of individual participant data).

It is not always easy to elicit expert opinion. There are difficulties in communicating the question and translating the experts' answers into parameters. With a systematic review including many studies, we would need expert opinion in each one of the studies and such a process would entail a large time burden. This was not our intention in this work as we placed more emphasis on establishing the statistical model. An easier solution is to conduct a thorough sensitivity analysis. We can start assuming $\delta_{\mathrm{ij}}^{\mathrm{imp}}=\delta_{\mathrm{ij}}^{\text{miss}}=0\forall i,j$ and start moving gradually away from the LOCF analysis by considering $\delta_{\mathrm{ij}}^{\mathrm{imp}}∼N\left(\mu_{\delta_{\mathrm{ij}}^{\mathrm{imp}}},\sigma_{\delta_{\mathrm{ij}}^{\mathrm{imp}}}^{2}\right)\;$and $\delta_{\mathrm{ij}}^{\text{miss}}∼N\left(\mu_{\delta_{\mathrm{ij}}^{\text{miss}}},\sigma_{\delta_{\mathrm{ij}}^{\text{miss}}}^{2}\right)\;$with increasingly larger values for mean values and standard deviations. The sensitivity analysis should be prespecified in the protocol analysis.

A simple approach would be to assume $\mu_{\delta_{\mathrm{ij}}^{\mathrm{imp}}}=\mu_{\delta_{\mathrm{ij}}^{\text{miss}}}=0$ and increasingly assume larger values for $\sigma_{\delta_{\mathrm{ij}}^{\mathrm{imp}}}^{2}$ and $\sigma_{\delta_{\mathrm{ij}}^{\text{miss}}}^{2}$. This would be ideal if one believes in LOCF as it surpasses the problem of having spuriously narrow confidence intervals.

## 5 ANALYSIS OF MOTIVATING EXAMPLE

**Figure A1:**
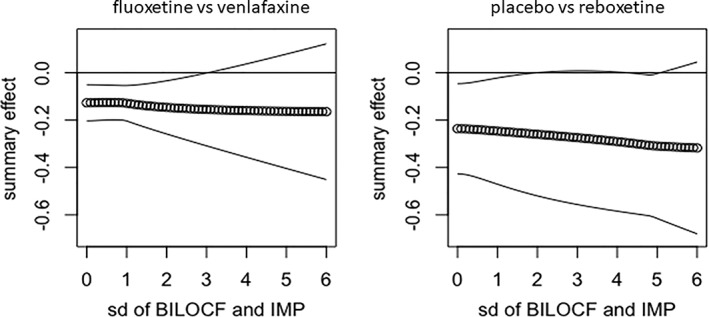
(Dotted line) summary effect and (solid lines) 95% confidence interval. The mean value of bias in the last observation carried forward (BILOCF) and informative missingness difference of means (IMDoM) is zero and their standard deviation is shown in the horizontal axis

We suggest assuming $\delta_{\mathrm{ij}}^{\mathrm{imp}}=\delta_{\mathrm{ij}}^{\text{miss}}=0$ as the primary analysis, which is equivalent to the LOCF analysis. Any difference in the mean values $\mu_{\delta_{\mathrm{ij}}^{\mathrm{imp}}}$ and $\mu_{\delta_{\mathrm{ij}}^{\text{miss}}}$ across groups would favor one treatment over the other. We take a neutral stance, assuming a zero mean for the BILOCF and IMDoM parameters in both groups ( $\mu_{\delta_{\mathrm{ij}}^{\mathrm{imp}}}=\mu_{\delta_{\mathrm{ij}}^{\text{miss}}}=0$). Most probably, this scenario is not realistic but we use it for illustration purposes. We let the standard deviation of the BILOCFs and the IMDoMs assume a range of values from 0 up to 6. The fact that we impute uncertainty around BILOCF and IMDoM would increase within‐study variation. Hence, the pooled effect would change because study effect sizes would be weighted differently. In this example, missing and imputation rates are similar across studies and we do not expect big fluctuations. Figure A1 in the Appendix shows the summary effect size, denoted by a solid line, and its 95% confidence limits, denoted by the dotted lines, under various scenarios with $\mu_{\delta_{\mathrm{ij}}^{\mathrm{imp}}}=\mu_{\delta_{\mathrm{ij}}^{\text{miss}}}=0$ and with increasing $\;\sigma_{\delta_{\mathrm{ij}}^{\mathrm{imp}}},\sigma_{\delta_{\mathrm{ij}}^{\text{miss}}}$ reflected in the horizontal axis. We assume that the BILOCF and IMDoM parameters are independent across arms and with each other and that $\sigma_{\delta_{\mathrm{ij}}^{\mathrm{imp}}}=\sigma_{\delta_{\mathrm{ij}}^{\text{miss}}}$. We made this choice so that we will not a priori favor either of the drugs. The summary effect is similar across the various scenarios with a minor reduction due to the different weights assigned to the studies. We observe that, when $\sigma_{\delta_{\mathrm{ij}}^{\mathrm{imp}}}=\sigma_{\delta_{\mathrm{ij}}^{\text{miss}}}>5$, ie, $\delta_{\mathrm{ij}}^{\mathrm{imp}}∼N\left(0,5^{2}\right)$ and $\delta_{\mathrm{ij}}^{\text{miss}}∼N\left(0,5^{2}\right)$, the summary estimate becomes nonsignificant for fluoxetine vs venlafaxine. In the comparison placebo vs reboxetine, results become nonsignificant instantly, ie, $\delta_{\mathrm{ij}}^{\mathrm{imp}}∼N\left(0,2^{2}\right)$ and $\delta_{\mathrm{ij}}^{\text{miss}}∼N\left(0,2^{2}\right)$, suggesting that even minor doubts about the LOCF results would result in no differences between the two groups. Table 5 shows the summary effect assuming various scenarios. Some scenarios are neutral in the sense that they assume that the distributions for BILOCF and IMDoM are the same across the two arms (scenarios N1‐N5); other nonneutral scenarios assume different distributions across the two arms so that either fluoxetine (F1‐F3) or venlafaxine is favored (V1‐V3). Neutral scenarios do not have a large impact on the results unless $\sigma_{\delta_{\mathrm{ij}}^{\mathrm{imp}}}=\sigma_{\delta_{\mathrm{ij}}^{\text{miss}}}>5$. In this case, there is a small drop in the summary effect because relative weights are reassigned and a study with a positive SMD (Keller, 2009) loses much of its weight (Table E1 in the Appendix). However, with that much uncertainty around IMDoM and BILOCF, the summary effect becomes nonstatistically significant. The Keller 2009 study is by far the largest in this meta‐analysis with 266 and 781 participants randomized to fluoxetine and venlafaxine, respectively (Table 2). It also has large imputation numbers (47 and 124), but their imputation rates are similar to those of other studies (Table 2). However, the penalty given to that study is relatively large exactly because of the large weight this study has on the LOCF analysis. Not trusting the LOCF results impacts mainly studies with large imputation rates. If imputation rates are similar across studies, not trusting the LOCF results impacts larger studies whose effect size has a larger impact on the summary results.

**Table 5:** Random‐effects meta‐analysis results for summary effect size (standardized mean difference [SMD]), 95% confidence intervals (CIs), and heterogeneity standard deviation for the two sources of evidence and for various BIP and IMP scenarios. Results from the sensitivity analyses are based on fourteen studies

	Scenarios	SMD (95% CI)
**Neutral Scenarios**		
	Complete case analysis [1]	−0.28 (−0.51,−0.04)
	LOCF analysis [2]	−0.13 (−0.20, −0.05)
N1	$\delta_{\mathrm{ij}}^{\mathrm{imp}}∼N\left(0,3^{2}\right)$, $\delta_{\mathrm{ij}}^{\text{miss}}∼N\left(0,3^{2}\right)$	−0.11 (−0.21, −0.02)
N2	$\delta_{\mathrm{ij}}^{\mathrm{imp}}∼N\left(0,5^{2}\right)$, $\delta_{\mathrm{ij}}^{\text{miss}}∼N\left(0,5^{2}\right)$	−0.12 (−0.24, −0.00)
N3	$\delta_{\mathrm{ij}}^{\mathrm{imp}}∼N\left(0,10^{2}\right)$, $\delta_{\mathrm{ij}}^{\text{miss}}∼N\left(0,10^{2}\right)$	−0.13 (−0.33, 0.07)
N4	$\delta_{\mathrm{ij}}^{\mathrm{imp}}∼N\left(-5,2^{2}\right)$, $\delta_{\mathrm{ij}}^{\text{miss}}∼N\left(5,2^{2}\right)$	−0.09 (−0.17, −0.01)
N5	$\delta_{\mathrm{ij}}^{\mathrm{imp}}∼N\left(-10,5^{2}\right)$, $\delta_{\mathrm{ij}}^{\text{miss}}∼N\left(10,5^{2}\right)$	−0.10 (−0.22, 0.03)
**Scenarios That Favor Fluoxetine**		
F1	$\delta_{\mathrm{iT}}^{\mathrm{imp}}∼N\left(-5,2^{2}\right)$, $\delta_{\mathrm{iC}}^{\mathrm{imp}}∼N\left(-10,2^{2}\right)$, $\delta_{\mathrm{iT}}^{\text{miss}}∼N\left(5,2^{2}\right)$, $\delta_{\mathrm{iC}}^{\text{miss}}∼N\left(10,2^{2}\right)$	0.00 (−0.09, 0.08)
F2	$\delta_{\mathrm{iT}}^{\mathrm{imp}}=0$, $\delta_{\mathrm{iC}}^{\mathrm{imp}}∼N\left(-5,2^{2}\right)$, $\delta_{\mathrm{iT}}^{\text{miss}}=0$, $\delta_{\mathrm{iC}}^{\text{miss}}∼N\left(5,2^{2}\right)$	−0.01 (−0.08, 0.07)
F3	$\delta_{\mathrm{iT}}^{\mathrm{imp}}∼N\left(-5,5^{2}\right)$, $\delta_{\mathrm{iC}}^{\mathrm{imp}}∼N\left(-10,5^{2}\right)$, $\delta_{\mathrm{iT}}^{\text{miss}}∼N\left(5,5^{2}\right)$, $\delta_{\mathrm{iC}}^{\text{miss}}∼N\left(10,5^{2}\right)$	−0.03 (−0.15, 0.10)
**Scenarios That Favor Venlafaxine**		
V1	$\delta_{\mathrm{iT}}^{\mathrm{imp}}∼N\left(-5,2^{2}\right)$, $\delta_{\mathrm{iC}}^{\mathrm{imp}}=0$, $\delta_{\mathrm{iT}}^{\text{miss}}∼N\left(5,2^{2}\right)$, $\delta_{\mathrm{iC}}^{\text{miss}}=0$	−0.19 (−0.28, −0.11)
V2	$\delta_{\mathrm{iT}}^{\mathrm{imp}}∼N\left(-10,2^{2}\right),$ $\delta_{\mathrm{iC}}^{\mathrm{imp}}∼N\left(-5,2^{2}\right)$, $\delta_{\mathrm{iT}}^{\text{miss}}∼N\left(10,2^{2}\right),$ $\delta_{\mathrm{iC}}^{\text{miss}}∼N\left(5,2^{2}\right)$	−0.17 (−0.25, −0.08)
V3	$\delta_{\mathrm{iT}}^{\mathrm{imp}}∼N\left(-10,5^{2}\right)$, $\delta_{\mathrm{iC}}^{\mathrm{imp}}∼N\left(-5,5^{2}\right)$, $\delta_{\mathrm{iT}}^{\text{miss}}∼N\left(10,5^{2}\right)$, $\delta_{\mathrm{iC}}^{\text{miss}}∼N\left(5,5^{2}\right)$	−0.18 (−0.31, −0.06)

[1] Complete case analysis is based on four studies.

[2] Last observation carried forward (LOCF) analysis is based on thirteen studies.

## 6 DISCUSSION

Missing data have not been handled properly in most trials, potentially leading to biased and overprecise results. These problems are propagated in a synthesis of trials through a meta‐analysis, and we run the risk of finding a false‐positive result because of the inflated sample sizes within trials. The LOCF method has been typically requested by regulatory agencies on the grounds that it is a conservative method, but this is mistaken and recommendations have been against its use.^1^, ^16^, ^17^, ^18^ In this paper, we focused on LOCF, but the methodology can be applied to other imputation schemes. Another well‐known method is baseline observation carried forward (BOCF) in which the outcome at the end of the study is replaced by its baseline measurement and is typically employed when patients withdrew from trials because of adverse events and LOCF is seen as insufficiently conservative.^19^, ^20^ This equal to assuming that missing participants have not improved/deteriorated at all.

Most depression trials report the outcome values from the LOCF analysis. We agree with the current practice that considers an LOCF analysis or a complete case analysis to be the primary analysis in a meta‐analysis. The suggested methodology can be used alongside as a sensitivity analysis. It is easily understood conceptually that, by using LOCF, we not only run the risk of getting a biased outcome but also artificially increase the sample size of the study. Missing data are usually MNAR. Participants may drop out of a study because they do not see any improvement or because of drug‐related side effects. Because drugs usually differ in terms of effectiveness and side effects, we expect different imputation rates and time points of dropout across the groups of a study. The method can easily extend to network meta‐analysis.^21^ We created R code^22^ (given in Appendix E) that uses Equations (11) and (12) to compute the adjusted effect sizes and standard errors and, then, uses R package “meta” to synthesize them.^23^ We have also created a Stata^24^ command that will become available through “mtm.uoi.gr” and would be an extension of the recently developed command metamiss2.^25^

It is not always straightforward how to embed an expert's beliefs into a statistical model. We may have data on intermediate time points that show a very different effect across time points. It could be the case, depending on the field, that there is a seemingly significant effect during the first weeks that is lost at the end of the trial (transient effect). In depression trials, this may be the case when placebo or a suboptimal treatment is involved. It is important that experts understand the reasons people drop out of a study group or collect reasons for dropout. If they leave with unequal rates, then missing data may well introduce bias. There may be bias in favor of the group with the highest imputation rate if participants are expected to deteriorate over time and in favor of the group with the lowest imputation rate if participants are expected to improve over time. The researcher may try to adjust results by making assumptions about the BILOCF parameter that favor the group that is not favored by the imputation rates. One way to inform the missing data parameters is through individual participant data (from the studies where it is available) or from trials in the systematic review that have results on all time points. In a comparison between two antidepressants, the one with the smallest imputation rate is favored as patients stay in the study longer with more chances of seeing any improvement.

Any analysis about missing data has to make untestable assumptions because the actual data needed to test the assumptions are missing. These assumptions can be used mathematically to inform effect estimates in a sensitivity analysis. Hence, starting with the LOCF analysis, we then consider various scenarios about the informative missingness parameters and explore how robust results are. The outcomes can be adjusted in such a wide range of ways there is a risk that one may, deliberately or not, make assumptions in favor of a certain drug. To minimize such a risk, we suggest that the sensitivity analyses should be prespecified and described in detail in the protocol and that values for the BILOCF and IMDoM parameters should be chosen on clinical grounds.

The validity of the analysis rests on the plausibility of the assumptions made. Clinicians with expertise in clinical trials have a good understanding of the reasons for missingness in clinical trials, but caution is needed in translating this expertise into values for the BILOCFs and the IMDoMs. We plan to continue working on how to formulate the appropriate questions to elicit information about the distributions of $\delta_{\mathrm{ij}}^{\mathrm{imp}}\;$and $\delta_{\mathrm{ij}}^{\text{miss}}$. Extra caution is needed when trying to elicit correlation parameters that are not easily understood by clinicians. Missing participants dropout for various reasons, and ideally, these reasons are reported. It may be unrealistic to assume the same BILOCF or IMDoM across all missing participants. In Appendix D, we present how one can assume different scenarios for the various types of missing participants.

Even if we do not wish to favor any of the interventions, we suggest assuming departures from the missing distribution assuming the same distributions for BILOCF and IMDoM across the groups of the study (neutral scenarios). An expert may inform us on which drug is likely to be favored by the LOCF analysis and consider nonneutral scenarios in the opposite drug.

In practice, it is very time consuming to define BILOCF and IMDoM for all studies taking into account their characteristics and some grouping is necessary (eg, all placebo control studies have the same BILOCF and IMDoM).

Another limitation of the model presented here is that we associated the mean outcome in the missing participants with the true outcome and not with the outcome reported in the trial. The reason we did this was to avoid potential contamination due to the LOCF imputed outcomes. However, experts might be more comfortable relating missing values to a quantity for which the data provides an estimate. The maths could be adapted to do this.

It is likely that dropouts in a randomized controlled trial would have dropped out in real life as well. Even in that case, LOCF would underestimate/overestimate a drug's efficacy if patients are expected to improve/deteriorate over time. The target in randomized controlled trials is to get an unbiased effect estimate at the predesignated primary outcome measurement point. Such an estimate would inform us about the true effectiveness of the experimental intervention. Dropouts and side effects should be taken into account (this is also why the dropout rate is a major outcome in depression trials) when informing the patient of the benefits and costs of each drug.

Our model suggests an extra source of variance (around imputed and missing outcome data). If studies have similar imputation/missing rates, then reweighting the studies would give more weight to small studies because we add an extra source of uncertainty that would have a relatively larger impact on large studies with small variances. This is similar to what is happening when we go from fixed to random effect meta‐analysis. Hence, we have to take into account how much confidence we would like to place to small and large studies. Small studies may be poorly reported (eg, not report missing data) and hence get overweighted in the suggested analysis.

## APPENDIX A

### CALCULATION OF IMPUTATION‐ADJUSTED VARIANCE

Using the result for the variance of the product of two independent random variables A and B

$$\text{var}\left(AB\right)=\left(E{\left(B\right)}^{2}+\text{var}\left(B\right)\right)\text{var}\left(A\right)+E{\left(A\right)}^{2}\text{var}\left(B\right)$$

with $\;A=\left(1-\pi_{\mathrm{ij}}^{*\mathrm{com}}\right)$ and $B=\delta_{\mathrm{ij}}^{\mathrm{imp}},$ we take the variance of the true mean outcome for those who provided some data (Equation (5) in the manuscript) as

$$V\left(\chi_{\mathrm{ij}}^{\mathrm{rep}}|\text{data}\right)\approx V\left({\tilde{\chi }}_{\mathrm{ij}}^{\mathrm{rep}}|\text{data}\right)+\frac{\left(\mu_{\delta_{\mathrm{ij}}^{\mathrm{imp}}}^{2}+\sigma_{\delta_{\mathrm{ij}}^{\mathrm{imp}}}^{2}\right)p_{\mathrm{ij}}^{*\mathrm{com}}p_{\mathrm{ij}}^{*\mathrm{imp}}}{n_{\mathrm{ij}}^{\mathrm{com}}+n_{\mathrm{ij}}^{\mathrm{imp}}}+p_{\mathrm{ij}}^{{*\mathrm{imp}}^{2}}\sigma_{\delta_{\mathrm{ij}}^{\mathrm{imp}}}^{2},$$

which is actually Equation (10) in the manuscript.

By taking the variance of Equation (8) conditional on the observed data using the above result for random variables *A* and *B*, we get

$$V\left(\chi_{\mathrm{ij}}^{\mathrm{tot}}|\text{data}\right)\approx V\left(\chi_{\mathrm{ij}}^{\mathrm{rep}}|\text{data}\right)+\frac{\left(\mu_{\delta_{\mathrm{ij}}^{\text{miss}}}^{2}+\sigma_{\delta_{\mathrm{ij}}^{\text{miss}}}^{2}\right)p_{\mathrm{ij}}^{\mathrm{rep}}p_{\mathrm{ij}}^{\text{miss}}}{n_{\mathrm{ij}}^{\mathrm{com}}+n_{\mathrm{ij}}^{\mathrm{imp}}+n_{\mathrm{ij}}^{\text{miss}}}+p_{\mathrm{ij}}^{{\text{miss}}^{2}}\sigma_{\delta_{\mathrm{ij}}^{\text{miss}}}^{2}.$$

Then, by using Equation (10) for $V\left(\chi_{\mathrm{ij}}^{\mathrm{rep}}|\text{data}\right)$, we get

$$V\left(\chi_{\mathrm{ij}}^{\mathrm{tot}}|\text{data}\right)\approx V\left({\tilde{x}}_{\mathrm{ij}}^{\mathrm{rep}}\right)+\frac{\left(\mu_{\delta_{\mathrm{ij}}^{\mathrm{imp}}}^{2}+\sigma_{\delta_{\mathrm{ij}}^{\mathrm{imp}}}^{2}\right)p_{\mathrm{ij}}^{*\mathrm{com}}p_{\mathrm{ij}}^{*\mathrm{imp}}}{n_{\mathrm{ij}}^{\mathrm{com}}+n_{\mathrm{ij}}^{\mathrm{imp}}}+p_{\mathrm{ij}}^{{*\mathrm{imp}}^{2}}\sigma_{\delta_{\mathrm{ij}}^{\mathrm{imp}}}^{2}+\frac{\left(\mu_{\delta_{\mathrm{ij}}^{\text{miss}}}^{2}+\sigma_{\delta_{\mathrm{ij}}^{\text{miss}}}^{2}\right)p_{\mathrm{ij}}^{\mathrm{rep}}p_{\mathrm{ij}}^{\text{miss}}}{n_{\mathrm{ij}}^{\mathrm{com}}+n_{\mathrm{ij}}^{\mathrm{imp}}+n_{\mathrm{ij}}^{\text{miss}}}+p_{\mathrm{ij}}^{{\text{miss}}^{2}}\sigma_{\delta_{\mathrm{ij}}^{\text{miss}}}^{2},$$

which is Equation (12) in the manuscript

## APPENDIX B

### COMPUTATION OF EFFECT SIZES FOR SIMPLE AND NETWORK META‐ANALYSIS

We would like to compute the adjusted effect size of Equation (13)

$$\;\beta_{i}=f\;\left(\chi_{iT}^{\text{tot}}\right)-f\;\left(\chi_{iC}^{\text{tot}}\right),$$

where *j* = *C* and *j* = *T* refer to the control and treatment group and *f* is a link function that determines the effect measure.

We would like to estimate *E*(*β*_*i*_| ***data***) and *V*(*β*_*i*_| ***data***).

For *E*(*β*_*i*_| ***data***), we have to estimate

$$E\;\left(\beta_{i}|\text{data}\right)=E(f\left(\chi_{\mathrm{iT}}^{\mathrm{tot}}|\text{data}\right)-E\;\left(f\left(\chi_{\mathrm{ic}}^{\mathrm{tot}}|\text{data}\right)\right).$$

For the variance of a sum of two random variables, it holds

$$\text{var}\left(A\pm B\right)=\text{var}\left(A\right)+\mathrm{var}\left(B\right)\pm 2\text{cov}\left(A,B\right).$$

Data are independent in the two arms. If we let the BILOCF and IMDoM parameters follow univariate normal distributions, ie, free such as $\delta_{\mathrm{ij}}^{\mathrm{imp}}∼N\;\left(\mu_{\delta_{\mathrm{ij}}^{\mathrm{imp}}},\sigma_{\delta_{\mathrm{ij}}^{\mathrm{imp}}}^{2}\right)$ and $\delta_{\mathrm{ij}}^{\text{miss}}\;∼\;N\;\left(\mu_{\delta_{\mathrm{ij}}^{\text{miss}}},\sigma_{\delta_{\mathrm{ij}}^{\text{miss}}}^{2}\right)$ or study‐specific such as $\delta_{i}^{\mathrm{imp}}\;∼\;N\;\left(\mu_{\delta_{i}^{\mathrm{imp}}},\sigma_{\delta_{i}^{\mathrm{imp}}}^{2}\right)\;$and $\delta_{i}^{\text{miss}}\;∼\;N\;\left(\mu_{\delta_{i}^{\text{miss}}},\sigma_{\delta_{i}^{\text{miss}}}^{2}\right)$, then there is no covariance term between $f\;\left(\chi_{\mathrm{iT}}^{\mathrm{tot}}|\text{data}\right)$ and $f\;\left(\chi_{\mathrm{ic}}^{\mathrm{tot}}|\text{data}\right)$.

We may however let $\delta_{\mathrm{ij}}^{\mathrm{imp}}$ and $\delta_{\mathrm{ij}}^{\text{miss}}$ be correlated across arms. For example, we may assume bivariate normal distributions with correlation $\rho_{\delta_{i}^{\mathrm{imp}}}$ and $\rho_{\delta_{i}^{\text{miss}}}$, respectively; that is, $\text{corr}\left(\delta_{\mathrm{iC}}^{\mathrm{imp}},\delta_{\mathrm{iT}}^{\mathrm{imp}}\right)=\rho_{\delta_{i}^{\mathrm{imp}}}$ and $\text{corr}\left(\delta_{\mathrm{iC}}^{\text{miss}},\delta_{\mathrm{iT}}^{\text{miss}}\right)=\rho_{\delta_{i}^{\text{miss}}}$ expressed as follows:

$$\left(\begin{array}{c} \delta_{iC}^{\text{imp}}\\ \delta_{iT}^{\text{imp}} \end{array}\right)∼N\left(\left(\begin{array}{c} \mu_{\delta_{ic}^{\text{imp}}}\\ \mu_{\delta_{iT}^{\text{imp}}} \end{array}\right),\left(\begin{array}{cc} \sigma_{\delta_{iC}^{\text{imp}}}^{2} & \rho_{\delta_{i}^{\text{imp}}}\sigma_{\delta_{iC}^{\text{imp}}}\sigma_{\delta_{iT}^{\text{imp}}}\\ \rho_{\delta_{i}^{\text{imp}}}\sigma_{\delta_{iC}^{\text{imp}}}\sigma_{\delta_{iT}^{\text{imp}}} & \sigma_{\delta_{iT}^{\text{imp}}}^{2} \end{array}\right)\right)$$

and

$$\left(\begin{array}{c} \delta_{iC}^{\text{miss}}\\ \delta_{iT}^{\text{miss}} \end{array}\right)∼N\left(\left(\begin{array}{c} \mu_{\delta_{iC}^{\text{miss}}}\\ \mu_{\delta_{iT}^{\text{miss}}} \end{array}\right),\left(\begin{array}{cc} \sigma_{\delta_{iC}^{\text{miss}}}^{2} & \rho_{\delta_{i}^{\text{miss}}}\sigma_{\delta_{iC}^{\text{miss}}}\sigma_{\delta_{iT}^{\text{miss}}}\\ \rho_{\delta_{i}^{\text{miss}}}\sigma_{\delta_{iC}^{\text{miss}}}\sigma_{\delta_{iT}^{\text{miss}}} & \sigma_{\delta_{iT}^{\text{miss}}}^{2} \end{array}\right)\right).$$

For estimating MDs, *f* is the identity function. The imputation‐adjusted mean outcome and variance are given in Equations (15) and (16) in the manuscript.

For estimating SMDs, we have $f\;\left(u_{i}\right)=\frac{u_{i}}{S_{i}}$, where $S_{i}=\sqrt{\frac{\left(n_{\mathrm{iT}}-1\right)s_{\mathrm{iT}}^{2}+\left(n_{\mathrm{iC}}-1\right)s_{\mathrm{iC}}^{2}}{n_{\mathrm{iT}}+n_{\mathrm{iC}}-2}}$ and the imputation‐adjusted mean outcome and variance are given by dividing Equations (15) and (16) in the manuscript with *S*_*i*_ and $S_{i}^{2}$, respectively.

So far, we assumed that $\delta_{\mathrm{ij}}^{\mathrm{imp}}$ and $\delta_{\mathrm{ij}}^{\text{miss}}$ are independent. If $\delta_{\mathrm{ij}}^{\mathrm{imp}}$ and $\delta_{\mathrm{ij}}^{\text{miss}}$ are not independent, they can be jointly modeled in a bivariate normal distribution. Then, we have to add to Equation (12) the quantity $2p_{\mathrm{ij}}^{*\mathrm{imp}}p_{\mathrm{ij}}^{\text{miss}}\sigma_{\delta_{\mathrm{ij}}^{\mathrm{imp}}}\sigma_{\delta_{\mathrm{ij}}^{\text{miss}}}\rho_{\delta_{\mathrm{ij}}^{\mathrm{imp}},\delta_{\mathrm{ij}}^{\text{miss}}}$, where $\rho_{\delta_{\mathrm{ij}}^{\mathrm{imp}},\delta_{\mathrm{ij}}^{\text{miss}}}$ is the correlation coefficient between $\delta_{\mathrm{ij}}^{\mathrm{imp}}$ and $\delta_{\mathrm{ij}}^{\text{miss}}.$

The method is easily extended to network meta‐analysis. If there are three‐arm trials, the correlation between effect sizes using a common comparator should be accounted for. Suppose that, in a three‐arm trial, we estimate *β*_*iAB*_ and *β*_*iAC*_. In this case, *β*_*i*_ = (*β*_*iAB*_, *β*_*iAC*_)^′^ follows a bivariate normal distribution with covariance given by the formula

$$\begin{array}{c} \text{cov}\left(\;\beta_{\mathrm{iAB}},\beta_{\mathrm{iAC}}\right)\approx \text{cov}\;\left(\chi_{\mathrm{iB}}^{\mathrm{tot}},\chi_{\mathrm{iC}}^{\mathrm{tot}}|\mathrm{dat}a_{i}\right)\chi_{\mathrm{iT}}^{\mathrm{tot}}\chi_{\mathrm{iC}}^{\mathrm{tot}}-\text{cov}\;\left(\chi_{\mathrm{iA}}^{\mathrm{tot}},\chi_{\mathrm{iB}}^{\mathrm{tot}}|\mathrm{dat}a_{i}\right)\chi_{\mathrm{iA}}^{\mathrm{tot}}\chi_{\mathrm{iB}}^{\mathrm{tot}}\\ -\text{cov}\;\left(\chi_{\mathrm{iA}}^{\mathrm{tot}},\chi_{\mathrm{iC}}^{\mathrm{tot}}|\mathrm{dat}a_{i}\right)\chi_{\mathrm{iA}}^{\mathrm{tot}}\chi_{\mathrm{iC}}^{\mathrm{tot}}+\mathrm{var}\;\left(\chi_{\mathrm{iA}}^{\mathrm{tot}}|\mathrm{dat}a_{i}\right)\chi_{\mathrm{iA}}^{\mathrm{tot}}\chi_{\mathrm{iA}}^{\mathrm{tot}}. \end{array}$$

If, instead of MD, we consider SMD, the above Equation should be multiplied by $\frac{1}{S_{i}^{2}}$, where $S_{i}^{2}$ is the pooled variance.

## APPENDIX C

### PRIOR ELICITATION

To inform the IMDoM parameter $\delta_{\mathrm{ij}}^{\mathrm{imp}}$, we inform the experts of the data we have observed and ask them their opinion about potential differences in the missing data. For example, suppose that we have a depression trial comparing reboxetine to placebo, just like in Example 2, and the outcome is measured in the HAMD scale. We may ask the experts the following question.

“*Suppose that XX% of patients allocated to reboxetine had completed the final interview and their mean score in the HAMD scale is 20 with standard deviation 6 (so that about 95% of these participants have values between 8 and 32). What is your expectation for the mean outcome score for those who did not provide any outcome data compared with those who completed the trial?”*

The experts are then asked to distribute a total weight of 100 across nine categories. Most likely, we would expect missing data to be judged worse than completers. In Table C1, we give an example of the answers of an expert who believes there is no difference between missing participants and completers, and the beliefs of a second expert who believes that missing participants did worse than completers.

**Table C1:** Eliciting expert opinion to evaluate the differences in the outcomes between missing and reported outcomes. Experts are asked to distribute a total of 100 across the nine categories. This table shows the responses of an expert who believes that there are no big differences between missing and reported outcomes, and an expert who believes that missing participants had worse outcomes than those reported

*The mean score in the HAMD scale of those who were randomized to reboxetine and completed the final questionnaire is 20 with standard deviation 6*									
*(so that about 95% of these participants have values between 10 and 30). What is your expectation for the mean outcome score for those who do not*									
*completed the final questionnaire, compared with those who completed it?*									
First expert	Interval of mean change for the noncompleters								
	Worse than reported outcomes by				Same as reported outcomes	Better than reported outcomes by			
	−12	−9	−6	−3		3	6	9	12
	around 8	around 11	around 14	around 17	around 20	around 23	around 26	around 29	around 32
Your answers	5	8	12	15	20	15	12	8	5
Second expert	Interval of mean change for the noncompleters								
Worse than reported outcomes by				Same as reported outcomes	Better than reported outcomes by			
	−12	−9	−6	−3		3	6	9	12
	around 8	around 11	around 14	around 17	around 20	around 23	around 26	around 29	around 32
Your answers	5	10	35	35	10	5	0	0	0

Abbreviations: HAMD, Hamilton Rating Scale for Depression.

## APPENDIX D

### DIFFERENT BILOCF PARAMETERS

We expect to observe different effects in those dropping out before 4 weeks after the onset of treatment and in those dropping out after 4 weeks. We introduce two BILOCFs, one referring to the period before 8 weeks and one to the period from 8 weeks onwards. Similarly, if we have *K* groups, we introduce *K* BILOCF parameters.

$$\begin{array}{c} \delta_{\mathrm{ij}}^{\mathrm{imp}\left(k\right)}=\chi_{\mathrm{ij}}^{\mathrm{imp}\left(k\right)}-{\tilde{x}}_{\mathrm{ij}}^{\mathrm{imp}\left(k\right)},\;k=1,\text{…},K\;\text{with}\\ \delta_{\mathrm{ij}}^{\mathrm{imp}\left(k\right)}∼N\;\left(\mu_{\delta_{\mathrm{ij}}^{\mathrm{imp}\left(k\right)}},\sigma_{\delta_{\mathrm{ij}}^{\mathrm{imp}\left(k\right)}}^{2}\right), \end{array}$$

where upper index *(k)* refers to the *k*th group. Hence, ${\tilde{\chi }}_{\mathrm{ij}}^{\mathrm{imp}}=\sum_{k=1}^{K}\pi_{\mathrm{ij}}^{*\mathrm{imp}\left(k\right)}{\tilde{x}}_{\mathrm{ij}}^{\mathrm{imp}\left(k\right)}\;$with $\pi_{\mathrm{ij}}^{*\mathrm{imp}}=\sum_{k=1}^{K}\pi_{\mathrm{ij}}^{*\mathrm{imp}\left(k\right)}{\tilde{x}}_{\mathrm{ij}}^{\mathrm{imp}\left(k\right)}$. Equation (5) is now written

$$\begin{array}{c} \chi_{\mathrm{ij}}^{\mathrm{rep}}=\pi_{\mathrm{ij}}^{*\mathrm{com}}\chi_{\mathrm{ij}}^{\mathrm{com}}+{\text{∑}}_{k=1}^{K}\pi_{\mathrm{ij}}^{*\mathrm{imp}\left(k\right)}\left(\delta_{\mathrm{ij}}^{\mathrm{imp}\left(k\right)}+{\tilde{x}}_{\mathrm{ij}}^{\mathrm{imp}\left(k\right)}\right)={\tilde{x}}_{\mathrm{ij}}^{\mathrm{obs}}+{\text{∑}}_{k=1}^{K}\pi_{\mathrm{ij}}^{*\mathrm{imp}\left(k\right)}\delta_{\mathrm{ij}}^{\mathrm{imp}\left(k\right)}, \end{array}$$

and Equations (9) and (10) are

$$E\;\left(\chi_{\mathrm{ij}}^{\mathrm{rep}}|\text{data}\right)={\tilde{x}}_{\mathrm{ij}}^{\mathrm{rep}}+{\text{∑}}_{k=1}^{K}\pi_{\mathrm{ij}}^{*\mathrm{imp}\left(k\right)}\mu_{\delta_{\mathrm{ij}}^{\mathrm{imp}\left(k\right)}}$$

and

$$V\;\left(\chi_{\mathrm{ij}}^{\mathrm{rep}}|\text{data}\right)=V\;\left({\tilde{\chi }}_{\mathrm{ij}}^{\mathrm{rep}}|\text{data}\right)+\sum_{k=1}^{K}\frac{\left(\mu_{\delta_{\mathrm{ij}}^{\mathrm{imp}\left(k\right)}}^{2}+\sigma_{\delta_{\mathrm{ij}}^{\mathrm{imp}\left(k\right)}}^{2}\right)\pi_{\mathrm{ij}}^{*\mathrm{com}}\pi_{\mathrm{ij}}^{*\mathrm{imp}\left(k\right)}}{n_{\mathrm{ij}}^{\mathrm{com}}+n_{\mathrm{ij}}^{\mathrm{imp}}}+\sum_{k=1}^{K}\pi_{\mathrm{ij}}^{{*\mathrm{imp}\left(k\right)}^{2}}\sigma_{\delta_{\mathrm{ij}}^{\mathrm{imp}\left(k\right)}}^{2}.$$

Similar logic can be applied when we believe that participants have dropped out for different reasons and we do not want one BILOCF parameter to express the difference between the true outcome $\chi_{\mathrm{ij}}^{\mathrm{imp}}$ and the imputed outcome ${\tilde{\chi }}_{\mathrm{ij}}^{\mathrm{imp}}$ in all patients who left the study early and were subsequently imputed with LOCF

## APPENDIX E

### R CODE

metamissLOCF < −

function (yT,sdT,cT,lT,mT,yC,sdC,cC,lC,mC,mgT,sdgT,mgC,sdgC,rhog,mlT,sdlT,mlC,sdlC,rhol, ES)

*{*

*### T denotes the treatment group and C denotes the control group*

*### y is the vector of effect sizes*

*### sd is the vector of standard deviations*

*### c is the vector with the number of completers per study*

*### l is the vector with the number of LOCF‐imputed values per study*

*### m is the vector with the number of missing outcome data per study*

*### mg and sdg refer to the mean value and standard deviation of the BIP distribution*

*### rhog refers to the correlation of the BIP random variables across groups*

*### ml and sdl refer to the mean value and standard deviation of the IMDoM distribution*

*### rhoL refers to the correlation of the IMDoM random variables across groups*

*##########probabilities for completers given observed##############*

*pcomC = cC/(cC + lC)*

*pcomT = cT/(cT + lT)*

*##########probabilities for observers##########*

*pobsC = (cC + lC)/(cC + lC + mC)*

*pobsT = (cT + lT)/(cT + lT + mT)*

*# Equation* (12) *for estimating the variance*

*vxcomC = sdC^2/(cC + lC) + (mgC^2 + sdgC^2)*pcomC*(1‐pcomC)/(cC + lC) + (1‐pcomC)^2*sdgC^2 + (mlC^2 + sdlC^2)*pobsC*(1‐pobsC)/(cC + lC + mC) + (1‐pobsC)^2*sdlC^2*

*vxcomT = sdT^2/(cT + lT) + (mgT^2 + sdgT^2)*pcomT*(1‐pcomT)/(cT + lT) + (1‐pcomT)^2*sdgT^2 + (mlT^2 + sdlT^2)*pobsT*(1‐pobsT)/(cT + lT + mT) + (1‐pobsT)^2*sdlT^2*

*##########effect size##########*

*# Equation* (11) *for estimating the expected value*

*xtotC = yC+(1‐pcomC)*mgC+(1‐pobsC)*mlC*

*xtotT = yT+(1‐pcomT)*mgT+(1‐pobsT)*mlT*

*##########variance for the effect size, including the correlation of IMDoM*

*covariance = rhol*sdlT*sdlC*(1‐pobsC)*(1‐pobsT) + rhog*sdgT*sdgC*(1‐pcomC)*(1‐pcomT)*

*#### choice of effect measure*

*if (ES==‘SMD’)*

*{*

*########## pooled standard deviation#####################*

*spooled = sqrt(((cC + lC‐1)*sdC^2 + (cT + lT‐1)*sdT^2))/sqrt (cC + cT + lC + lT‐2)*

*es = (xtotT‐xtotC)/spooled*

*var_es = (vxcomC + vxcomT‐2*covariance)/spooled^2*

*}*

*else*

*{*

*es = (xtotT‐xtotC)*

*var_es = vxcomC + vxcomT‐2*covariance*

*}*

*results = metagen (es,sqrt (var_es))*

*list (results$TE.random,results$lower.random,results$upper.random,results$tau)*

*}*

**Table E1:** Relative weights of studies from a random‐effects meta‐analysis under various scenarios

id	Weights From LOCF	Weights From Scenario N1	Weights From Scenario N2
	Analysis	$\delta_{\mathrm{ij}}^{\mathrm{imp}}∼N\left(0,3^{2}\right)$	$\delta_{\mathrm{ij}}^{\mathrm{imp}}∼N\left(0,5^{2}\right)$
		$\delta_{\mathrm{ij}}^{\text{miss}}∼N\left(0,3^{2}\right)$	$\delta_{\mathrm{ij}}^{\text{miss}}∼N\left(0,5^{2}\right)$
Clerc 1994	2%	3%	4%
Dierick 1996	10%	9%	8%
Tylee 1997	9%	12%	14%
Costaesilva 1998	12%	14%	15%
Alves 1999	3%	4%	4%
Rudolph 1999	6%	6%	6%
Silverstone 1999	8%	6%	5%
Tzanakaki 2000	3%	5%	6%
Schatzberg 2006	6%	5%	5%
Nemeroff 2007	6%	8%	9%
Keller 2007	25%	18%	13%
Sheehan 2009	6%	6%	6%
Heller 2009	1%	1%	1%
Chang 2015	4%	5%	5%

Abbreviations: LOCF, last observation carried forward.
